# A new Tie1 targeted antibody blocks tumor cell extravasation and metastasis

**DOI:** 10.15252/emmm.202012355

**Published:** 2020-05-13

**Authors:** Kabir A Khan, Robert S Kerbel

**Affiliations:** ^1^ Biological Sciences Platform Sunnybrook Research Institute Toronto ON Canada; ^2^ Department of Medical Biophysics University of Toronto Toronto ON Canada

**Keywords:** Cancer, Immunology, Vascular Biology & Angiogenesis

## Abstract

Targeting the metastatic process is a critical pursuit in the treatment of malignant disease. There are currently no specific anti‐metastatic drugs approved for clinical use, despite metastasis being the leading cause of death for cancer patients. Targeting the Tie1 receptor was shown as a possible strategy for selective anti‐metastasis therapies based on previous gene deletion studies. This current study is the first description of a human antibody against Tie1 with the potential for clinical use in targeting extravasation of tumor cells into organs such as the lung, without having a detrimental effect on immune cell infiltration.

## Graphical abstract

K. Khan and R. Kerbel highlight the first description of a human antibody against Tie1 with the potential for clinical use in targeting extravasation of tumor cells into organs such as the lung, without having a detrimental effect on immune cell infiltration.

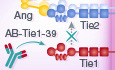

There are two well‐known angiogenesis‐regulating systems: the VEGF/VEGF receptor pathway and the angiopoietin (Ang)/Tie pathway. The targeting of the former has led to successes in treating cancer and vision loss conditions, whereas similar clinical successes have not yet been realized by targeting the Ang/Tie pathway. Tie1 and Tie2 are tyrosine kinase receptors expressed by endothelial cells (ECs) and some types of hematopoietic cells (Saharinen *et al*, 2017). Tie2 binds growth factors Ang1 and Ang2, whereas Tie1 does not; however, Tie1 can modulate Tie2 signaling through Tie1/Tie2 heterodimers. The Ang/Tie signaling axis is complex but generally speaking Ang1‐Tie2 interactions result in stabilization of newly formed vessels, whereas Ang2‐Tie2 interactions lead to vessel destabilization, effects which are context‐dependent. Therapies targeting this pathway have mainly focused on inhibiting the vascular plasticity promoting function of Ang2, where antibody‐blocking approaches can increase lung vessel integrity and decrease metastatic burden (Mazzieri *et al*, 2011; Holopainen *et al*, 2012). However, such Ang2 targeting agents have not yet had clinical success when used alone and are currently being investigated for use in combination with VEGF or immune checkpoint targeting drugs.

Tie1 upregulation in the tumor vasculature has been known for over 25 years (Kaipainen *et al*, 1994) but has only recently been investigated as a potential therapeutic target. Induced postnatal EC deletion of Tie1 in mice results in impaired primary tumor growth due to vascular defects and also leads to reduced metastasis due to inhibition of tumor cell extravasation (D'Amico *et al*, 2014; La Porta *et al*, 2018). Such genetic studies provided a basis for investigating Tie1 as a therapeutic target.

In this issue of *EMBO Molecular Medicine,* Singhal, Gengenbacher, and La Porta *et al* describe the generation of a Tie1‐targeted function‐blocking antibody that can inhibit metastasis (Singhal *et al*, 2020) (Fig 1). The authors undertook a novel approach in screening of Tie1‐blocking antibodies by using Tie2 signaling as a readout upon stimulation with Ang1. While the majority of antibodies agonized Tie1 and enhanced Tie2‐mediated Akt phosphorylation, one antibody was found to block this effect. This antibody, named AB‐Tie1‐39, was used in all subsequent experiments. In postnatal retinal angiogenesis assays, the antibody reduced angiogenesis, decreased the number of endothelial tip cells, and increased EC apoptosis. The AB‐Tie1‐39 antibody also reduced breast (4T1) and lung (LLC) mouse primary tumor growth, but did not cause significant disruptions in the tumor vasculature. This suggests that blocking of Tie1 using this antibody may be detrimental during developmental but not tumor angiogenesis, unlike genetic deletion of Tie1 in ECs, both of which are impaired (La Porta *et al*, 2018).

**Figure 1:**
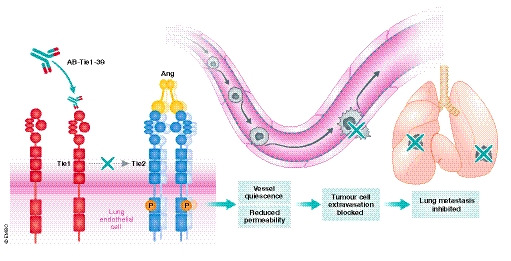
AB‐Tie1‐39 mechanism of action AB‐Tie1‐39 specifically binds to Tie1 which secondarily leads to increasing levels of Tie2 phosphorylation in lung endothelial cells. This results in vessel quiescence and reductions in vessel permeability which leads to disturbed extravasation of tumor cells, therefore inhibiting lung metastasis formation.

The most potent effects of AB‐Tie1‐39 were observed on lung metastases when tested in the neoadjuvant (i.e., before primary tumor resection) treatment setting in both 4T1 and LLC mouse tumor models, where metastatic burden was reduced. This was shown to be due, in all likelihood, to inhibiting tumor cell extravasation, on the basis of mice receiving AB‐Tie1‐39 pre‐treatment 7 days before intravenous injection of B16F10 melanoma cells, which led to markedly reduced lung metastasis. This was further confirmed by AB‐Tie1‐39 causing reduced *in vitro* tumor cell transmigration through an EC monolayer. Indeed, when the antibody was used in an adjuvant treatment setting (i.e., after primary tumor resection) and when tumor cell extravasation had likely already taken place, the antibody treatment had no effect on metastatic burden or overall survival. These data recapitulated previous findings involving genetic deletion of Tie1 after primary tumor resection (La Porta *et al*, 2018). The authors went on to further dissect the mechanism of AB‐Tie1‐39 and showed that in contrast to its Tie2 phosphorylation‐blocking effects *in vitro*, the antibody actually led to increased Tie2 phosphorylation in lung ECs *in vivo*. Tie1 has previously been shown to act contextually in an inhibiting or stimulating manner on Tie2 (Savant *et al*, 2015), and Tie2 phosphorylation can either lead to EC quiescence or angiogenesis depending on whether Tie2 is expressed at cell–cell or cell–matrix contacts (Fukuhara *et al*, 2008; Saharinen *et al*, 2008). The authors suggest that lung ECs, upon treatment with AB‐Tie1‐39, are in a more quiescent state, which was corroborated by microarray analyses of lung ECs revealing reduction in expression of genes related to EC migration and tumor cell adherence. A potential caveat for therapies that block tumor cell extravasation is the possibility of also interrupting immune cell trafficking into the tumor microenvironment, a major concern given the increasing impact of immunotherapy to treat cancer. In this regard, the authors showed that AB‐Tie1‐39 treatment did not result in significant differences in intra‐tumoral accumulation of a range of leukocytes, including T cells, dendritic cells, and macrophages, among others.

Therapeutic blocking of Tie1 could have wider uses in other diseases such as atherosclerosis, diabetic retinopathy, and liver fibrosis. In particular, endothelial‐specific Tie1‐deficient mice display less atherosclerosis in comparison with wild‐type mice when crossed with ApoE knockout mice which are highly susceptible to atherosclerosis (Woo *et al*, 2011). The newly described Tie1 ligand leukocyte cell‐derived chemotaxin 2 (LECT2) has been implicated in liver fibrosis where LECT2 binding to Tie1 results in dephosphorylation of Tie1, disruption of Tie1/Tie2 heterodimers, and promotion of Tie2/Tie2 homodimers, leading to enhanced liver fibrosis (Xu *et al*, 2019). Whether AB‐Tie1‐39 can block LECT2‐Tie1 binding or could be used as a therapy in liver fibrosis or indeed atherosclerosis may be interesting avenues of investigation.

Whether Tie1 is expressed on other cell types within the tumor microenvironment and what effects the antibody would have on such cells will also need to be investigated. Furthermore, whether Tie1 blockade could have effects on other sites of metastasis including liver and brain are important questions that need to be investigated. Neoadjuvant therapy is often performed in order to shrink a primary tumor and facilitate more successful surgical resection. However in this case, the neoadjuvant therapy may only reduce tumor burden modestly despite potentially having a profound inhibitory effect on subsequent metastases. Clinical trials will need to be designed with this in mind. Alternatively, AB‐Tie1‐39 could be combined with other therapies in the neoadjuvant setting to cause more robust tumor shrinkage, although such combination therapies could affect the efficacy of the antibody. In summary, this study presents a potentially clinically relevant anti‐metastatic agent that could have real‐world benefits, especially as a neoadjuvant therapy.

## Conflict of interest

RSK is a member of the Scientific Advisory Boards of Novelty Nobility, Seoul, CSTS Healthcare, Toronto, and OncoHost, Haifa and a recipient of a sponsored research agreement with Genentech, San Francisco. He is also a consultant to Novelty Nobility, CSTS Healthcare and Pharmabcine (Korea). KAK declares no conflict of interest.
